# Supplementary figures and images for: A mechanistically novel peptide agonist of the IL-7 receptor that addresses limitations of IL-7 cytokine therapy
Source: PLoS One. 2023 Oct 24;18(10):e0286834. doi: 10.1371/journal.pone.0286834 (PMC10597491; doi:10.1371/journal.pone.0286834)

## Slide 1
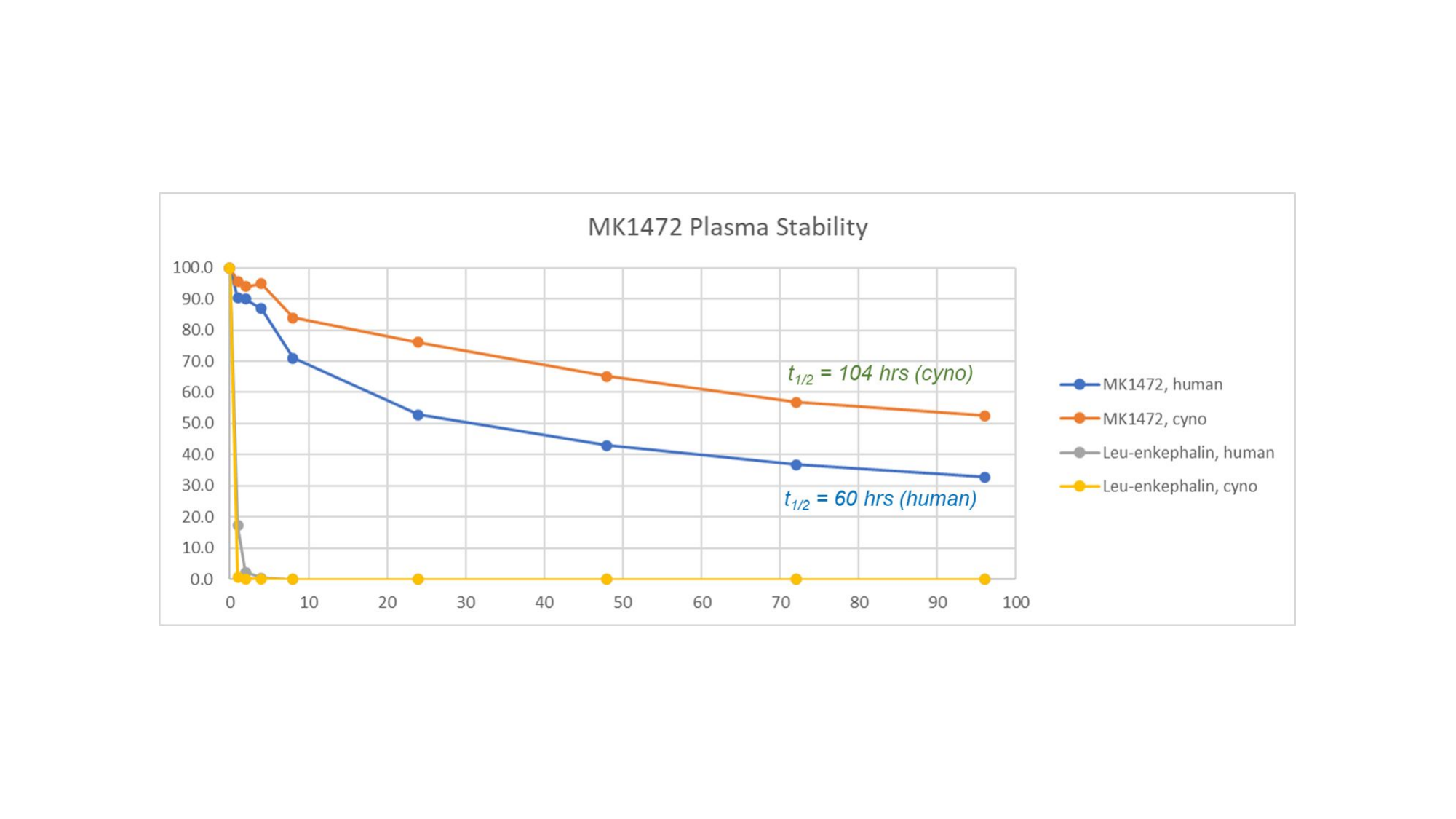

Supplement: S1 Fig — 10 mM MDK1472 or control peptide YGGFL incubated in duplicate in human or cynomolgus plasma at 37°C. At indicated time points, compounds were isolated from the plasma and the quantity of intact peptides measured by LC/MS/MS (Study performed by Quintara Discovery, Hayward, CA). (PPTX) [file pone.0286834.s001.pptx]

## Slide 1
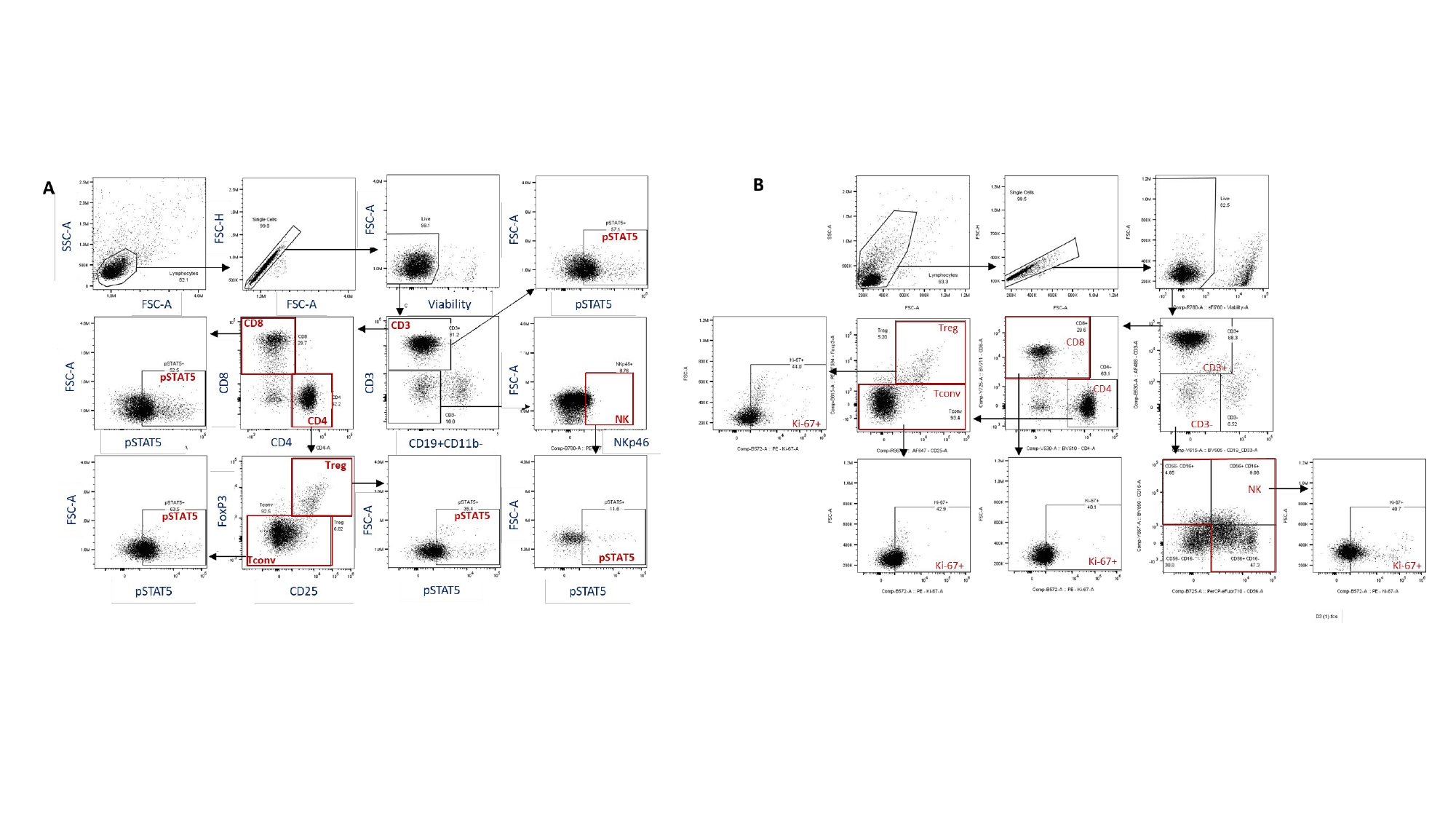

Supplement: S2 Fig — (A) pSTAT5 assay. (B) Ki-67 assay. (PPTX) [file pone.0286834.s002.pptx]

## Slide 1
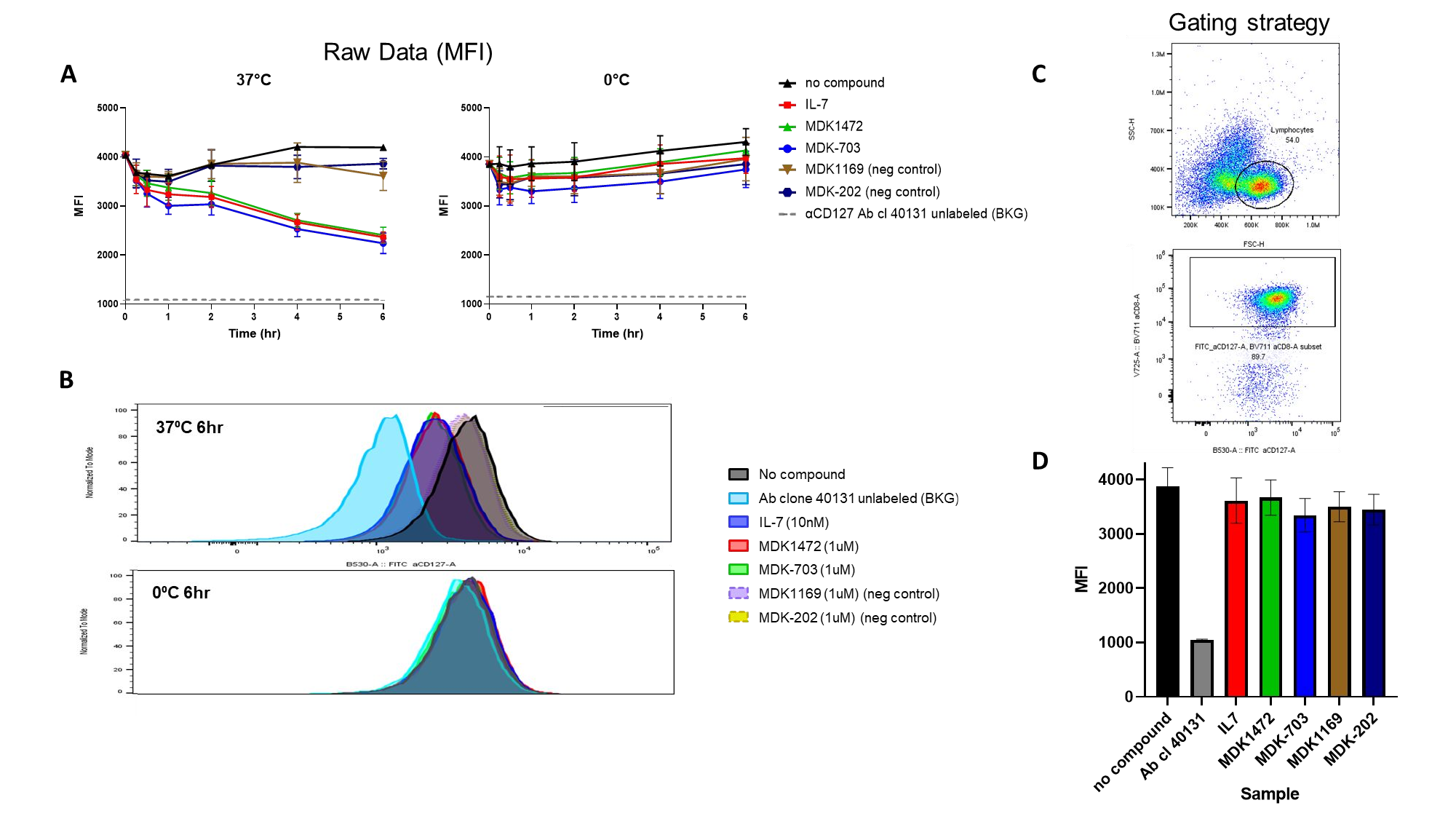

Supplement: S3 Fig — Primary MFI data (A) plotted time course, (B) histograms, (C) gating scheme. (D) Evaluation of interference of IL-7 and IL-7Rα agonist test compounds, and irrelevant test compounds with detection of cell surface IL-7Rα on cell surface by labelled anti-CD127 Ab clone #40131. All test compounds are loaded on the cells on ice for 35 min, stained, and analyzed by flow (as described in detail in Methods). (PPTX) [file pone.0286834.s003.pptx]

## Slide 1
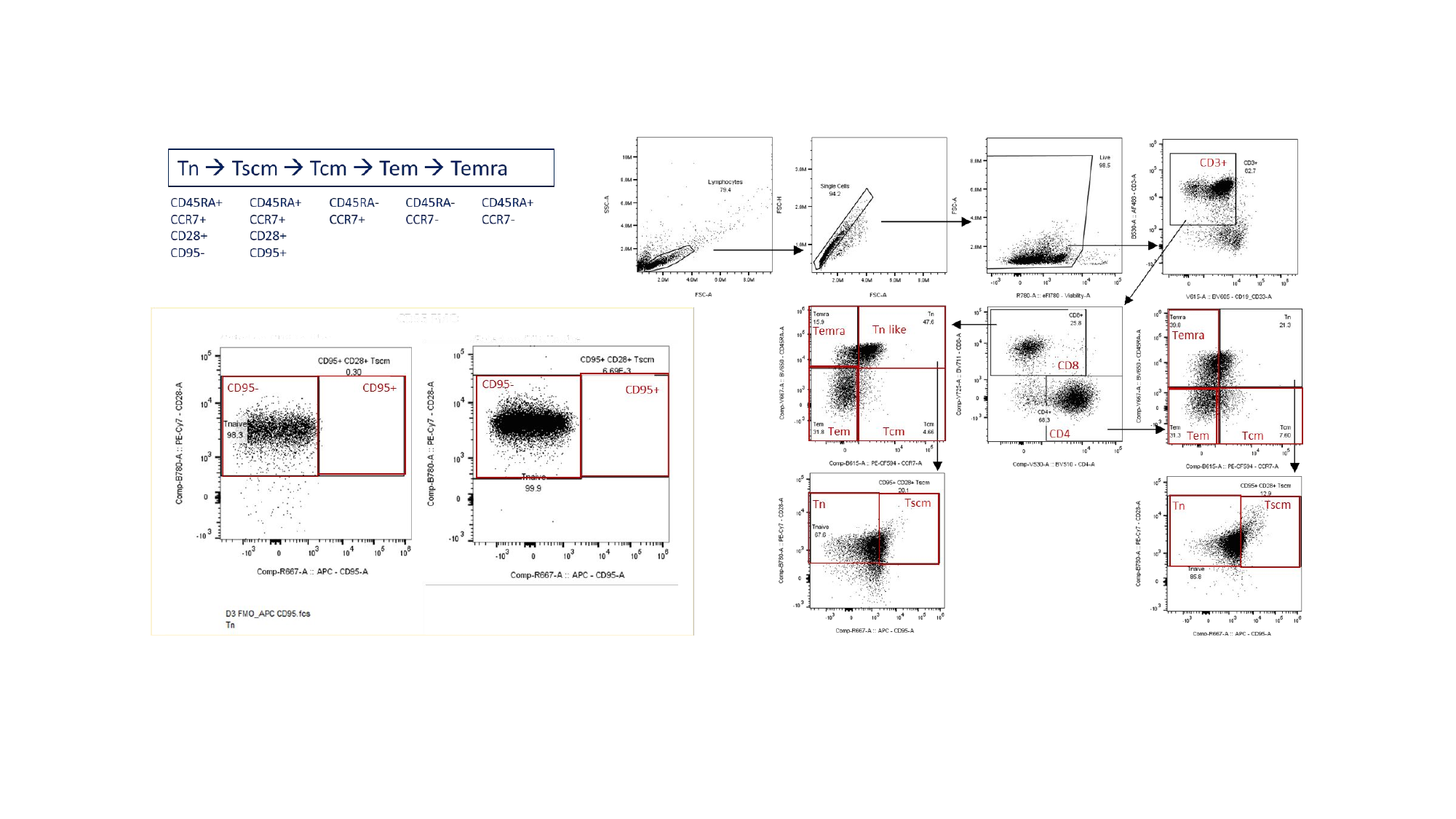

Supplement: S4 Fig — (PPTX) [file pone.0286834.s004.pptx]

## Slide 1
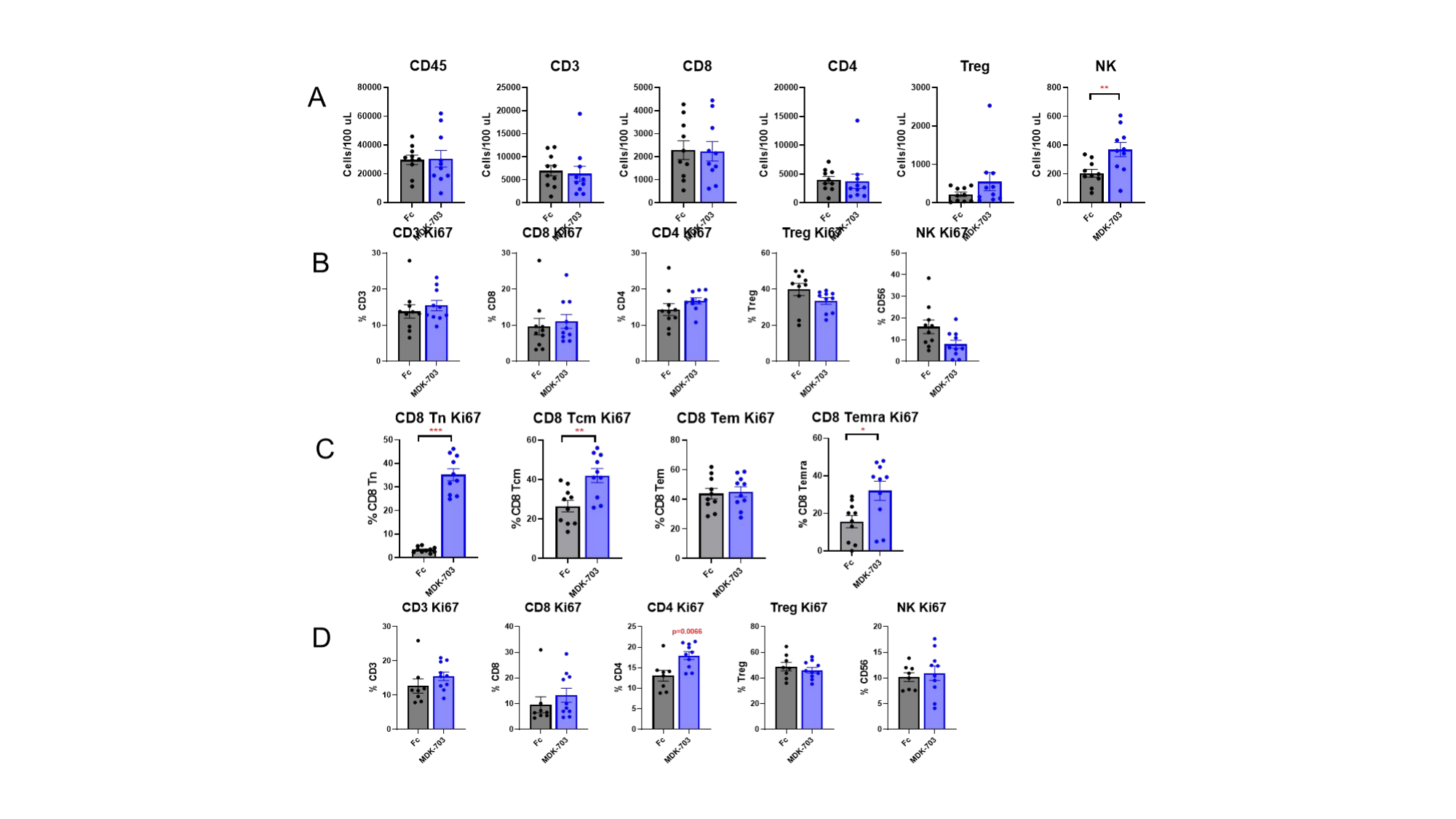

Supplement: S5 Fig — (A) Day 7 cell counts in blood. (B) Day 12 Ki-67 in blood. (C) Day 7 memory T-cells Ki-67 in blood. (D) Day 12 Ki-67 in spleen. (PPTX) [file pone.0286834.s005.pptx]

## Slide 1
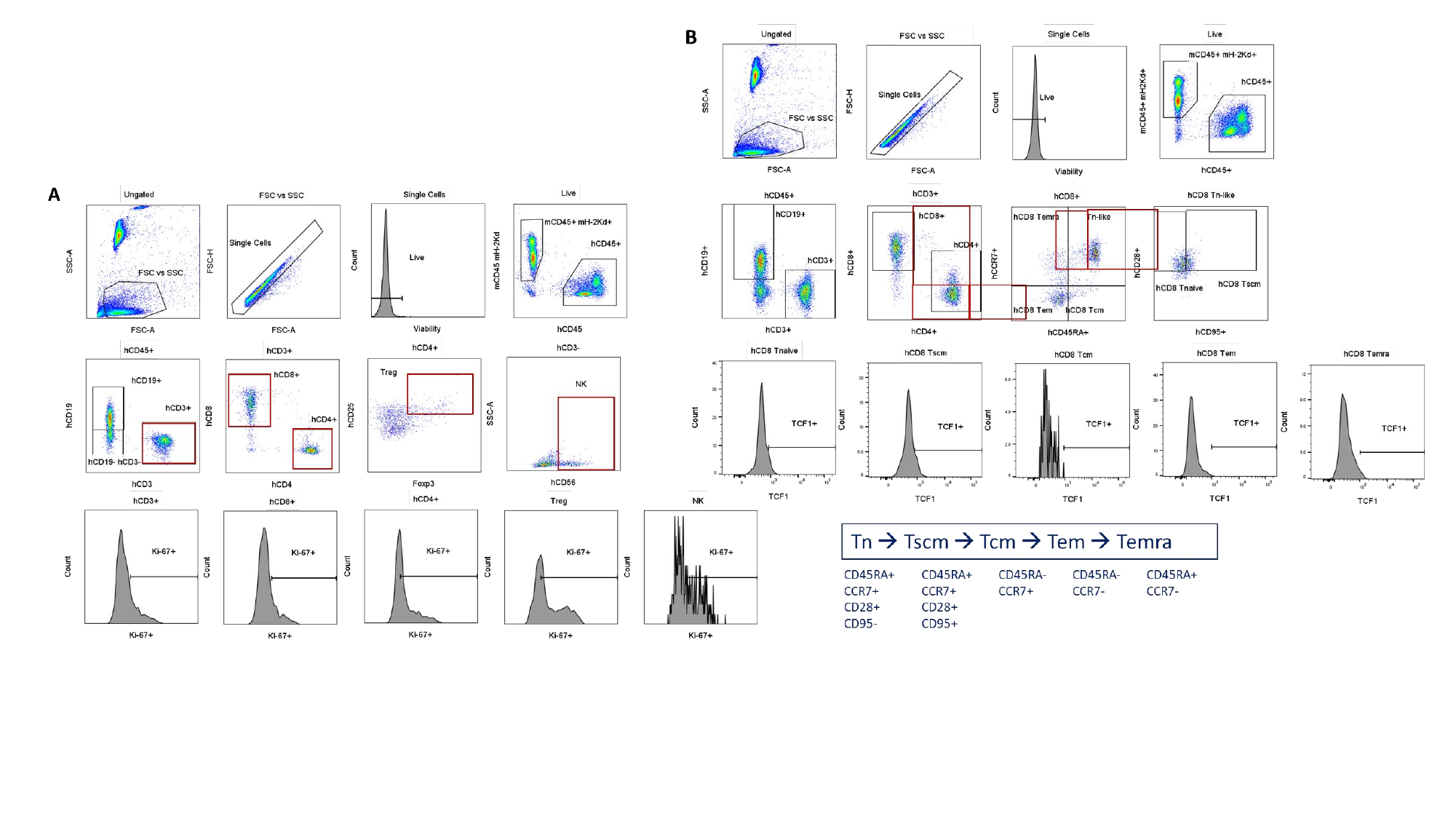

Supplement: S6 Fig — (A) TBNK Ki-67+. (B) Tn and Tmem TCF1+. (PPTX) [file pone.0286834.s006.pptx]

**S1 table. Potency values**

**
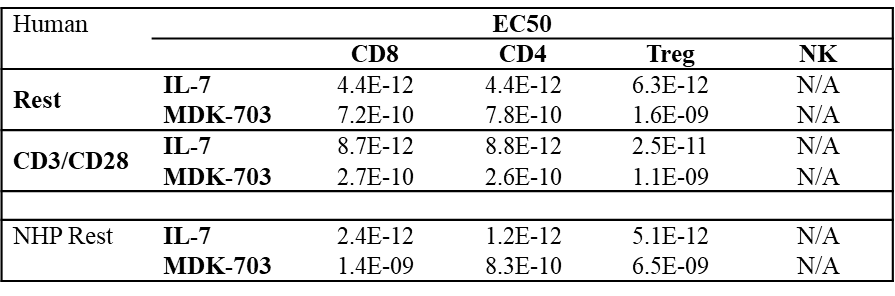
**

Supplement: S1 Table — (DOCX) [file pone.0286834.s007.docx]
