# Supplementary material for: A mechanistically novel peptide agonist of the IL-7 receptor that addresses limitations of IL-7 cytokine therapy
Source: PLoS One. 2023 Oct 24;18(10):e0286834. doi: 10.1371/journal.pone.0286834 (PMC10597491; doi:10.1371/journal.pone.0286834)
Supplement: S2 Table — (DOCX) [file pone.0286834.s008.docx]

**Table S2.**

| **Protein Name** | **UniProt Accession Number** |
| --- | --- |
| Human IgG2 Fc (MDK-703 expression construct) | P01859 |
|  |  |
| Human IL-2Rβ | P14784 |
| Human IL-4Rα | P24394 |
| Human IL-7Rα | P16871 |
| Human IL-9Rα | Q01113 |
| Human IL-15Rα | Q13261 |
| Human IL-21Rα | Q9HBE5 |
| Human CRLF2 transcript variant 1 Gene ORF cDNA clone expression plasmid (TSLPR) | Q9HC73 |
|  |  |
| Human IL-2 | P60568 |
| Human IL-4 | P05112 |
| Human IL-7 | P13232 |
| Human IL-9 | P15248 |
| Human IL-15 | P40933 |
| Human IL-21 | Q9HBE4 |
|  |  |
| Cynomolgus IL7Rα | Q38IC7 |
| Cynomolgus IL-7 | A0A2K5W745 |
