# Supplementary material for: A mechanistically novel peptide agonist of the IL-7 receptor that addresses limitations of IL-7 cytokine therapy
Source: PLoS One. 2023 Oct 24;18(10):e0286834. doi: 10.1371/journal.pone.0286834 (PMC10597491; doi:10.1371/journal.pone.0286834)
Supplement: S3 Table — (DOCX) [file pone.0286834.s009.docx]

**S3 Table. Labelled antibody reagents for flow cytometry**

| **Antibody** | Fluorochrome | Clone | Company | Catalog number |
| --- | --- | --- | --- | --- |
| **CCR7** | PE-CF594 | 150503 | BD Biosciences | 562381 |
| **CCR7** | PE/Cy7 | G043H7 | BioLegend | 353226 |
| **CD3** | AF488 | SP34.2 | BD Biosciences | 557705 |
| **CD3** | AF700 | SP34.2 | BD Biosciences | 557917 |
| **CD3** | BUV395 | SK7 | BD Biosciences | 564001 |
| **CD4** | BV510 | SK3 | BD Biosciences | 562970 |
| **CD4** | BV650 | L200 | BD Biosciences | 563737 |
| **CD4** | APC | SK3 | BioLegend | 344614 |
| **CD8** | BV711 | SK1 | BioLegend | 344734 |
| **CD8** | BUV737 | SK1 | BD Biosciences | 612755 |
| **CD8** | PE | BW135/80 | Miltenyi | 130-113-158 |
| **CD8** | PE-Cy7 | SK1 | BioLegend | 344712 |
| **CD8** | PE-eFluor 610 | RPA-T8 | eBioscience | 61-0088-42 |
| **CD11b** | BV605 | M1/70 | BioLegend | 101257 |
| **CD127** | PE | 40131 | R&D Systems | FAB306P |
| **CD159a** | PE-Cy7 | BAB281 | Beckman Coulter | A60797 |
| **CD16** | BV650 | B73.1 | BD Biosciences | 740639 |
| **CD19** | BV605 | HIB19 | BioLegend | 302244 |
| **CD19** | BV711 | HIB19 | BioLegend | 302246 |
| **CD20** | PE-Vio770 | LT20 | Miltenyi | 130-113-375 |
| **CD25** | AF647 | M-A251 | BioLegend | 356128 |
| **CD25** | AF780 | CD25-4E3 | ThermoFisher | 47-0257-42 |
| **CD25** | BB700 | M-A251 | BD Biosciences | 566447 |
| **CD25** | APC-eFluor780 | CD25-4E3 | Invitrogen | 47-0257-42 |
| **CD25** | BV510 | M-A251 | BioLegend | 356120 |
| **CD27** | PerCP-Cy5.5 | O323 | BioLegend | 302820 |
| **CD28** | PE-Cy7 | CD28.2 | BioLegend | 302926 |
| **CD33** | BV605 | P67.6 | BioLegend | 366612 |
| **CD45** | AF488 | HI30 | BioLegend | 304017 |
| **CD45** | BV785 | HI30 | BioLegend | 304048 |
| **CD45RA** | BV650 | HI100 | BioLegend | 304135 |
| **CD45RO** | BV421 | UCHL1 | BioLegend | 304224 |
| **CD56** | PerCP-eFluor 710 | CMSSB | Invitrogen | 46-0567-42 |
| **CD56** | BV605 | HCD56 | BioLegend | 318334 |
| **CD95** | APC | DX2 | BD Biosciences | 558814 |
| **FoxP3** | PE-CF594 | 236A/E7 | BD Biosciences | 563955 |
| **FoxP3** | PE | 206D | BioLegend | 320108 |
| **FoxP3** | PE | PCH101 | Invitrogen | 12-4776-42 |
| **FoxP3** | PE | 259D | eBioscience | 12-4776-42 |
| **Ki67** | PE | SolA15 | Invitrogen | 12-5698-82 |
| **Ki67** | BV421 | B56 | BD Biosciences | 562899 |
| **Ki67** | PerCP/Cy5.5 | Ki-67 | BioLegend | 350520 |
| **Live/Dead** | eFl780 | - | Invitrogen | 65-0865-18 |
| **Live/Dead** | Pacific Orange | - | Invitrogen | L34967 |
| **mCD45** | FITC | 30-F11 | BioLegend | 103108 |
| **mH-2Kd** | FITC | SF1-1.1 | BioLegend | 116605 |
| **NKp46** | PE-Cy7 | BAB281 | Beckman Coulter | B38703 |
| **pSTAT5** | BV421 | 47/Stat5 (pY694) | BD Biosciences | 562984 |
| **TCF-1** | AF488 | S33-966 | BD | 567018 |
